# Distilling experience into a physically interpretable recommender system for computational model selection

**DOI:** 10.1038/s41598-023-27426-5

**Published:** 2023-02-08

**Authors:** Xinyi Huang, Thomas Chyczewski, Zhenhua Xia, Robert Kunz, Xiang Yang

**Affiliations:** 1 Department of Mechanical Engineering, Pennsylvania State University, University Park, PA 16802 USA; 2 Department of Engineering Mechanics, Zhejiang University, Hangzhou, 310027 Zhejiang China

**Keywords:** Fluid dynamics, Mechanical engineering

## Abstract

Model selection is a chronic issue in computational science. The conventional approach relies heavily on human experience. However, gaining experience takes years and is severely inefficient. To address this issue, we distill human experience into a recommender system. A trained recommender system tells whether a computational model does well or poorly in handling a physical process. It also tells if a physical process is important for a quantity of interest. By accumulating this knowledge, the system is able to make recommendations about computational models. We showcase the power of the system by considering Reynolds-averaged-Navier–Stokes (RANS) model selection in the field of computational fluid dynamics (CFD). Since turbulence is stochastic, there is no universal RANS model, and RANS model selection has always been an issue. A working model recommending system saves fluid engineers years and allows junior CFD practitioners to make sensible model choices like senior ones.

## Introduction

Computational science has to rely on modeling since directly solving governing equations is often too costly. This is particularly true in fluid dynamics. Engineers have to rely on tools like Reynolds averaged Naver Stokes (RANS) and their associated turbulence models. Because of the stochastic nature of turbulence, there is not (and won’t be) a universal RANS model^1,2^. Hence, RANS model selection has always been a practical issue. The conventional approach to this issue is very direct. One would pick a benchmark flow, conduct RANS analysis with a number of available RANS models, select the best model, and use that model for flows that are similar^3^.

While the above methodology has given us many insights into RANS modeling, its shortcomings are quite obvious: there are many RANS models, and it is not possible to quantify *a priori* the best RANS model for a given application. This situation has been exacerbated by the onset of machine learning tools that have led to numerous RANS model variants^4–7^. The obvious solution is to gather the experiences of many RANS practitioners. This has been done in the form of online databases and offline workshops^8,9^. The basic expectation is that a RANS practitioner can examine the data and get insights into different RANS models. The difficulty, however, is that there is too much data for any individual to distill into actionable model choices. The above situation presents a big data challenge^10,11^. We attempt to address this challenge by distilling human experiences into a recommender system. The objective is that, by making use of a trained recommender system, a junior engineer can make informed decisions with the same reliability as a much more experienced senior engineer, and a senior engineer could gain insights into RANS models with which they are unfamiliar.

**Figure 1:**
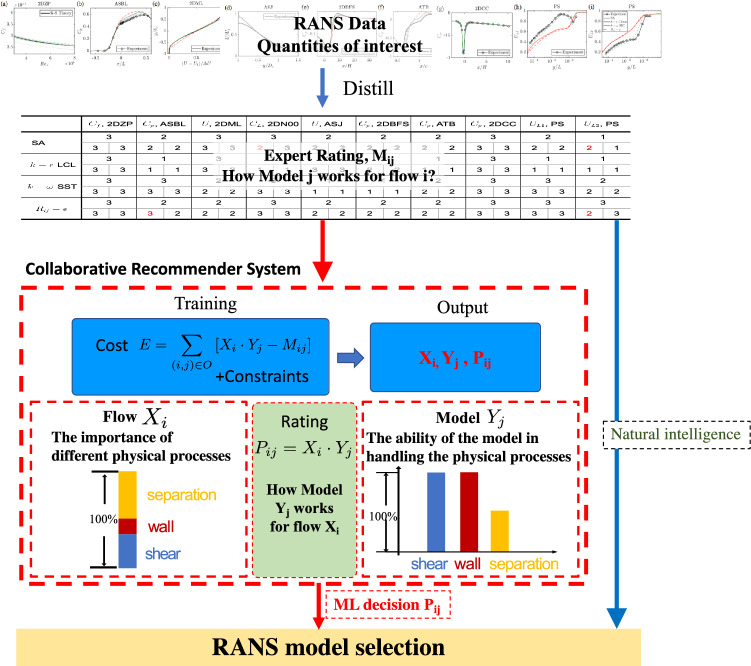
A sketch of the decision making process. The proposed approach is as follows. First we generate expert rating of the RANS results, $M_{ij}$, which describes how well model *j* works for flow *i*.. Second, we apply collaborative filtering to train a recommender system. The recommender system will learn how much different physical processes control flow *i*, i.e., $X_i$, and how well model *j* handles these physical processes, i.e., $Y_j$. The trained recommender system will predict a rating $P_{ij}=X_i\cdot Y_j$, which describes how model *j* works for flow *i*. Finally, a decision can be made using $P_{ij}$.

We explain how this can be done. First, we give a brief introduction to recommender systems. A recommender system is a subclass of information filtering systems that seeks to predict the “rating” a user would give to an item^12^. The rating measures how well an item suits a user’s needs. Recommender systems are used in generating playlists for video and music services and recommending content for social media platforms^13^. Consider, e.g., a recommender system for movies. The recommender system assigns each viewer (user) a “feature vector”. The feature vector describes the viewer’s preferences for movies. For example, the feature vector may be [action, horror, romance], and [1, 0, 0] corresponds to a viewer that likes action movies but dislikes horror and romance movies. The recommender system also assigns each movie (item) a feature vector. That vector describes the movie’s genre classification, again, [action, horror, romance]. A vector [0,0,1] corresponds to a movie with no action or horror elements but a lot of romance elements. A recommender system learns these vectors from viewers’ ratings. These ratings are not necessarily all “accurate”, but individual adversarial ratings can be filtered out when given enough data. The trained recommender system then generates a high rating when a viewer’s vector and a movie’s vector match well and a low rating when the two do not. The two vectors do not match in the above example, and the recommendation system assigns a low rating. It should be clear from the above discussion that a recommender system is not a black box. The parameters in a recommender system, i.e., the components in the users’ feature vectors and the items’ feature vectors, have clearly defined meanings.

We now make an analogy between movie recommendations and RANS model selection. We may regard RANS models as items and flow QoIs as users. A rating measures how well a RANS model works for a flow QoI. A QoI’s feature vector measures the importance of different physical processes. A RANS model’s feature vector measures how well the model handles the physical processes in the vector. A RANS model will work well when its feature vector matches that of a QoI’s. If we have two QoIs with similar feature vectors, a RANS model will have similar performance for both QoIs, i.e., similar ratings. If we have two models with similar feature vectors, they will have similar performance for the same QoI, too.

Given the similarities between movie recommendation and RANS model selection, we attempt to address the problem of RANS model selection via a recommender system, as illustrated in Fig. 1. Specifically, expert-rated RANS results (human experience) will be used as training data to train a recommender system. The training objective is to learn what physical processes are important to a flow and how well a RANS model handles these physical processes, i.e., to distill human experience into the recommender system.

**Figure 2:**
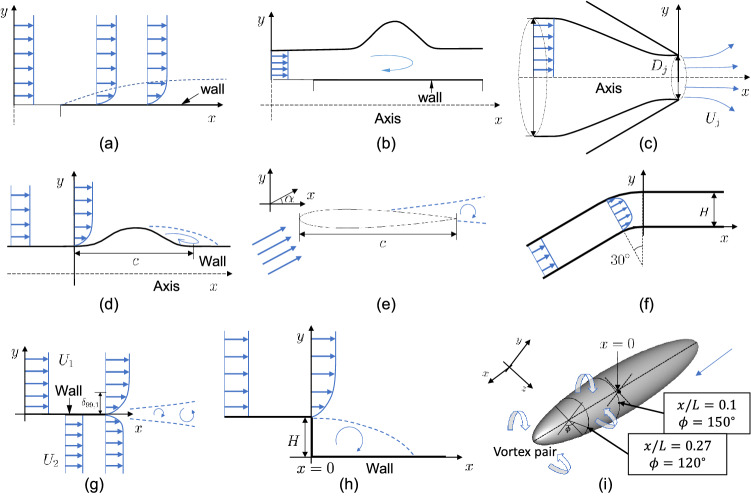
Flow schematics. (**a**) 2DZP, (**b**) ATB, (**c**) ASJ, (**d**) ASBL, (**e**) 2DN00, (**f**) 2DCC, (**g**) 2DML, (**h**) 2DBFS, and (**i**) PS. The basic flow phenomenology is sketched. The reader is directed to^36^ and^37^ for further details of these flows.

In this work, three recommender systems are trained separately for three RANS codes, namely, the in-house code NPHASE-PSU^14^, the commercial code STAR-CCM+^15^, and the open-source code TMR CFL3D^16^, respectively, to ensure the robustness of the method. Nine flows are considered. Figure 2 shows the sketches of the flows. They are a zero-pressure gradient boundary layer (2DZP), an axis-symmetric separated turbulent boundary layer (ASBL), a two-dimensional mixing layer (2DML), a two-dimensional NACA 0012 hydrofoil (2DN00) at various angles of attack, an axis-symmetric subsonic jet (ASJ), a back-facing step (2DBFS), an axisymmetric transonic bump (ATB), a two-dimensional convex boundary layer (2DCC) and a 6:1 prolate spheroid at $20^\circ$ angle of attack (PS), all of which have been extensively used for validation and verification. We consider the following 10 arbitrarily picked quantities of interest (QoIs): the wall skin friction coefficient $C_f$ for 2DZP, the wall pressure coefficient $C_p$ for ASBL, the velocity profile in the mixing layer for 2DML, the lift coefficient $C_L$ for 2DN00 at angles-of-attack $\alpha =0^\circ$, $10^\circ$, and $15^\circ$, the axial velocity profile in the jet for ASJ, the wall pressure coefficient $C_p$ for 2DBFS, ATB, and 2DCC, two velocity profiles at the indicated locations for PS. Table 1 shows the rating for all RANS models for STAR-CCM+. These ratings are generated by ranking the model results and will be our training data. Note that we do not necessarily have the ratings for every pair of RANS model and QoI, especially when the data is collected from the public database, such as TMR CFD3D data given in the supplementary Table S1.

**Table 1:** Ratings of all RANS models for all QoIs.

	$C_f$ 2DZP		$C_p$ ASBL		*U* 2DML		$C_L$ 2DN00		*U* ASJ		$C_p$ 2DBFS		$C_p$ ATB		$C_p$ 2DCC		$U_{L1}$ PS		$U_{L2}$ PS	
SA	3		2		3		3		2		2		2		3		2		1	
	3	3	2	2	3	3	2	3	2	2	2	2	2	2	3	3	2	2	2	1
$k-\epsilon$ LCL	3		1		3		1		3		2		1		3		1		1	
	3	3	1	1	3	3	1	1	3	3	2	2	1	1	3	3	1	1	1	1
$k-\omega$ SST	3		3		2		3		1		1		2		3		3		2	
	3	3	3	3	2	2	3	3	1	1	1	1	2	2	3	3	3	3	2	2
$R_{ij}-\epsilon$	3		2		2		3		2		2		2		3		3		3	
	3	3	3	2	2	2	3	3	2	2	2	2	2	2	3	3	3	3	2	3

The STAR-CCM+ results are shown here. The NPHASE-PSU results and the CFL3D results are given in supplementary Table 1. Here, 3 is good, 2 is fair, and 1 is poor. The number in the top row is the training data, and the two numbers in the bottom row are the predictions. The first predicted rating is by the baseline recommender system, and the second is by the augmented system. We color the predictions red if they are different from the data.

## Results

### Baseline recommender system

We show the results for the recommender system trained for STAR-CCM+. To keep the problem size manageable, four representative RANS models are considered, namely, the Spalart Allmaras (SA) model, the $k-\omega$ SST model, the $k-\epsilon$ LCL model, and the Reynolds stress model (which we denote as $R_{ij}-\epsilon$). The SA model is a one-equation eddy viscosity model. The $k-\omega$ SST and the $k-\epsilon$ LCL models are two-equation eddy viscosity models. The Reynolds stress model is a seven-equation model. It solves transport equations for all Reynolds stresses. We refer to this recommender system as the baseline recommender system (BRS). The results for the other two recommender systems trained for NPHASE-PSU and CFL3D are similar and are included in the supplemental material. Three physical processes are considered, i.e., F1 free shear, F2 wall, and F3 flow separation. The number of physical processes we are able to consider is limited by the amount of data. The system overfits if limited data is used to train too many physical processes. We follow the previous authors^17^ and regard flow separation as one issue/phenomenon but note that it is a simplification. The recommender system learns the QoIs’ feature matrix $\textbf{X}$ and the models’ feature matrix $\textbf{Y}$. $X_{in}$ measures how much process *n* controls the *i*th QoI. $Y_{jn}$ measures how well model *j* handles physical process *n*. Here, $i=1$, 2, ..., 10 because 10 QoIs are considered; $j=1$, 2, 3, 4 since 4 RANS models are assessed; $n=$1, 2, 3 as there are three physical processes. Hereon, we use *N* to denote the number of physical processes. In order to keep the same physical interpretation of feature vectors, we initialize the feature vectors by existing knowledge, detailed in the section “Methods”. Therefore, the training will converge the feature vectors to the nearest minimum thereby maintaining their original meanings. By definition, $\sum _{n=1}^N X_{in}=1$ for all *i* because a QoI is controlled by the *N* physical processes; $0<Y_{jn}<1$, where $Y_{jn}=0$ implies that the *j*th model does not handle process *n* at all, and $Y_{jn}=1$ implies that the *j*th model handles process *n* very well.

**Figure 3:**
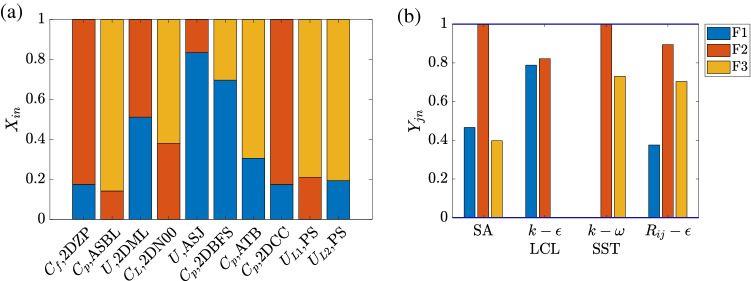
A visualization of (**a**) QoIs’ feature vectors and (**b**) models’ feature vectors.

Figure 3 shows the learned feature vectors for all 10 QoIs. Recall that the only information the recommender system has access to is the rating in Table 1. It has no access to the flow sketches in Fig. 2. Also, while an experienced fluid engineer may be able to tell how important the three physical processes are by examining the flow configuration in the sketch, the recommender system has no *a priori* fluid dynamics knowledge. Here, we compare the results in Fig. 3 and the sketches in Fig. 2. We can claim success if the learned feature vectors are physical. For brevity, we will use the notation [*Q*, *F*] when referring to the QoI *Q* in the flow *F*.

The recommender system shows that F3 flow separation is the dominant physical process for [$U_{L1}$, PS] and [$U_{L2}$, PS]. This is physical: the high angle of attack gives rise to massive separation, which will greatly affect the velocities near the suction side. The recommender system also considers flow separation important for [$C_p$, 2DBFS], [$C_p$, ASBL], [$C_p$, ATB] and [$C_L$, 2DN00], but not for [$C_f$, 2DZP], [*U*, 2DML], [*U*, ASJ], and [$C_p$, 2DCC]. This is physical as well: flow separates in 2DBFS, ASBL, ATB, and 2DN00, but not in 2DZP, 2DML, ASJ, and 2DCC. In addition to flow separation, F2 wall partly controls the following QoIs: [$C_f$, 2DZP], [*U*, 2DML], [$C_L$, 2DN00], [$C_p$, 2DCC], and [$U_{L1}$, PS], where a solid boundary is present; F1 free shear partly controls the following QoIs: [$C_f$, 2DZP], [*U*, 2DML], [*U*, ASJ], [$C_p$, 2DBFS], [$C_p$, 2DCC], and [$U_{L2}$, PS], where a shear layer is present. In all, we may conclude that the recommender system learns accurately what physical processes control a QoI from the training rating data.

Having examined the QoIs’ feature vectors *X*, we now examine the RANS models’ feature vectors *Y*, i.e., how well RANS models handle different physical processes. Such knowledge is usually gained through extensive validation and verification. The recommender system has no access to that literature. Even if we give the recommender system access to that literature, training a machine learning system to read and extract the needed information is a challenging task. Again, the only information the recommender system has is the ratings in Table 1. The results are shown in Fig. 3b. We will compare the results to the physical understanding we gained from the literature. SA is found to be insufficient for F3 flow separation. This agrees with the recent literature^18^, where the off-the-shelf SA model over predicts flow separation in a wing-body junction flow. The seven-equation Reynolds stress model $R_{ij}-\varepsilon$, on the other hand, handles F3 flow separation relatively well. This also agrees with the literature^19^, where the Reynolds stress model outperforms the two-equation SST model in predicting skin friction in a separated flow. Furthermore, comparing the $k-\omega$ SST model and the $k-\epsilon$ LCL model, we see that the former captures F3 flow separation much better than the latter, which is now a common knowledge^20^. Last but not least, all models handle F2 wall very well. This again agrees with the literature: flat-plate boundary layer is a case that all RANS model developers consider. Hence, we conclude that the recommender system learns how well a RANS model handles the physical processes.

**Figure 4:**
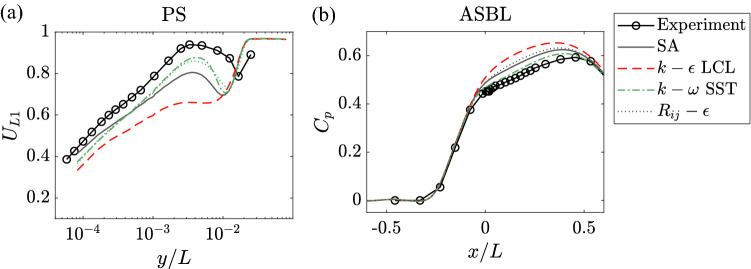
RANS results and the experimental data. Different lines are used for the results of different RANS models. Thin lines: SA model, dashed lines: $k-\epsilon$ LCL model, dot-dashed lines: $k-\omega$ SST model, and dotted lines: $R_{ij}-\epsilon$ model. The RANS results are color coded. Green: “good” results, gray: “fair” results, and red: “poor” results. (**a**) PS results, (**b**) ASBL results.

We now make use of the trained recommender system, i.e., the information contained in the $\textbf{X}$ and $\textbf{Y}$ matrices, to make predictions about whether a RANS model is going to work well for a flow. The general method is to examine if a QoI’s feature vector aligns well with a model’s feature vector. If they do, the model is considered to be able to handle the physical processes that are important to the QoI, and the model will work well for the QoI. If they do not, the model does not handle the physical processes that are important to the QoI, and the model won’t work well. For example, consider the $k-\epsilon$ model and [$U_{L1}$, PS]. We see that [$U_{L1}$, PS] is controlled mostly by F3 flow separation, which is not at all handled well by the $k-\epsilon$ model. Hence, these two feature vectors do not align well, and a low rating is predicted. The first number in the second row in Table 1 shows the predictions. We see that the trained recommender system gives very accurate ratings for the RANS models. To put these ratings in context, we compare some of the RANS results to data in Fig. 4. According to Table 1, the $k-\omega$ model and the Reynolds stress model work well for [$U_{L1}$, PS] but the $k-\epsilon$ model does not work well. This bears out in Fig. 4a, where the $k-\omega$ and $R_{ij}-\epsilon$ results follow the experimental measurements closely, whereas the $k-\epsilon$ result does not. The $k-\epsilon$ model is also rated poor for ASBL in Table 1, which also agree with the results in Fig. 4b. Errors are found in the rating of the Reynolds stress model for ASBL. The recommender system overrated the Reynolds stress model. This error is due to a limited number of physical processes considered in the recommender system. In the following sections, we expand the number of physical processes and train a larger recommender system.

Here, we note that the other two recommender systems trained for NPHASE-PSU and CFL3D do not have exactly the same feature vectors (see supplementary Figure S1). However, the minor differences do not lead to many changes in the predictions. The consistency of the predictions indicates the robustness of the recommender system.

### An augmented system

The size of a recommender system is mainly determined by the number of physical processes considered, i.e., *N*. Increasing *N* increases the risk of overfitting but also increases the potential predictive power of the recommender system (if there is enough data to train the recommender system). In the baseline case, *N* is 3, and F1 flow shear, F2 wall, and F3 flow separation are the physical processes. This is an over-simplification. For one, flow separation is not *a* process: it may well be broken down to, e.g., adverse pressure gradient, surface curvature, surface roughness, etc. For another, many physical processes that may control a QoI are not considered, e.g., flow stratification and system rotation.

Here, we increase *N* and study its impacts on the recommender system. It only makes sense to consider physical processes that are present in the data. For example, since none of the flows is stratified, including flow stratification as an additional physical process would not make sense. If we do, the recommender system would not learn anything meaningful. Here, the additional physical process we consider is pressure gradient, and the four physical processes are F1 flow shear, F2 wall, F3 pressure gradient, and F4 flow separation.

**Figure 5:**
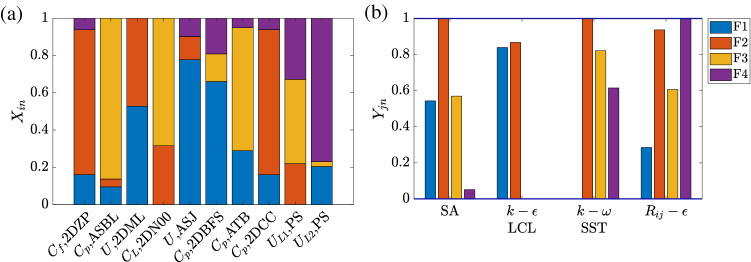
Same as Fig. 3 but for $N=4$.

The recommender system is re-trained for STAR-CCM+ data, and we refer to the trained recommender system as an augmented recommender system (ARS). Before we proceed to present the results, we first compute the number of parameters in the recommender system as a function of *N*, *I*, and *J*. The matrix $\textbf{X}$ has $I\times N-I$ parameters, here $-I$ is because the constraint $\sum _n X_{in}=1$. The matrix $\textbf{Y}$ has $J\times N$ parameters. It follows that BRS has $10\times 3-10+5\times 3=35$ parameters and ARS has $10\times 4-10+5\times 4=50$ parameters. Hence, increasing *N* from 3 to 4, the number of parameters increases from 35 in BRS to 50 in ARS. Considering that there are only 40 data points for STAR-CCM+, it is probably anticipated that ARS suffers from overfitting. According to Refs.^21,22^, the ratio between the number of parameters and the number of the training data is a good indicator of whether the system will overfit. Increasing the parameter number will lead to an overly optimistic accuracy. The exact number of data points needed is case-specific. As a rule of thumb, if the number of parameters is comparable to or less than the number of the data points, the model tends to be stable and this mitigates the risk of overfitting. It may be worth noting that many machine learning models suffer from uncontrolled errors due to overfitting. For example, Ling et al.^6^ trained a neural network that contains 5 neurons in the input layer, 30 neurons in 8 hidden layers, and 10 neurons in the output layer. The number of parameters in the neural network is close to 7000, yet the training data is from 2 flows, and the testing data is from 1 flow.

We show the results for STAR-CCM+. Again, the reader is directed to the supplement material for NPHASE and CFL3D results. The ARS predicted model ratings are listed in Table 1 as well. Compared to BRS, ARS is more “accurate”. The recommender system learns about the additional physical process from data and now gives more accurate ratings for QoIs that are controlled by the pressure gradient, e.g., [$U_{L2}$, PS] and [$C_L$, 2DN00]. It also accurately rates $R_{ij}-\epsilon$’s [$C_p$, ASBL], where an adverse pressure gradient leads to flow separation.

Figure 5 shows the QoIs’ feature vectors, and the models’ feature vectors. Pressure gradient is found to have a role in [$C_p$, ASBL], [$C_L$, 2DN00], [$C_p$, 2DBFS], [$C_p$, ATB], [$U_{L1}$, PS] in ARS, where the flow is subjected to a pressure gradient. Besides F3 pressure gradient, F4 flow separation is still the most important physical process for [$U_{L1}$, PS] and [$U_{L2}$, PS], and the Reynolds stress model is still the best in terms of handling F4 flow separation. Both make sense and agree with the baseline results. However, we can also find errors due to overfitting. First, F2 wall becomes one of the dominating effects for [*U*, 2DML], which does not cause trouble for ratings, but is not physical (see Fig. 2). Also, albeit small, F4 flow separation shows up in [*U*, ASJ] and [$C_f$, 2DZP], where there is no flow separation.

### Intended applications

A recommender system is able to learn from model ratings (and model ratings alone) the physical processes that control a flow and a model’s ability to capture these processes. This knowledge can be accumulated and passed from one agent (person, company, institution) to another agent, enabling more sensible model choices among multiple agents. The nature of matrix factorization allows the possibility of transfer learning, and thus the augmentation of the system without redoing the training. The trained system can then tell us if a RANS model will work well for a previously modeled flow for which we know the controlling physical process. Here, the RANS model result does not necessarily need to exist for that flow as long as we know the model’s ability to handle the controlling processes. To predict if an existing RANS model will work well for a new flow which has not been seen by the recommender system, we need the flow’s feature vector. The guess of the flow’s feature vector can be obtained by inspecting the flow configuration like in Fig. 2. The process requires only heuristics. For example, it is straightforward that the skin friction in case BFS is controlled mostly by separation and not so much by wall and free shear.

Last, if a new model is developed, this system can be used to determine its capability. We can use the new model to compute various flows (this is usually done by the developed when a new is developed) and rate the results. These results will then help us learn the model’s feature vector, which describes the model’s capability.

## Discussion

Computational modeling is an active research area that generates a constant stream of new models. Recently, applications of machine learning in computational science add to that stream, significantly increasing the number of available models. The number of new models is overwhelming, and it is not always clear which model one should employ for a problem. Hence, model selection is a pressing practical problem. This is particularly true for RANS model selection in the field of CFD. The problem is conventionally dealt with through extensive validation and verification, which encompasses model uncertainty quantification^23^, Bayesian weighing among models^24,25^, and tailoring a model for a particular application^10^, to name a few. This is inefficient. This work attempts to solve the problem of computational model selection via a recommender system. We consider RANS model selection in CFD. We show that a recommender system learns from data like a human. Specifically, it learns and parameterizes how well a RANS model handles a physical process and how much a QoI is controlled by a physical process. A trained recommender system rates a RANS model for a QoI: when the RANS model is able to handle the physical processes that control the QoI, a high rating is given, and vice versa. Last, we note that while this work shows a lot of promise, more can be done at a much larger scale, making use of all available RANS data and considering all available RANS models—a work that is left for future investigation.

## Methods

### Recommender systems

Consider *I* QoIs and *J* RANS models. The *i*th QoI’s feature vector is $\textbf{X}_{i}$, and $X_{in}$ is the *n*th component in $\textbf{X}_{i}$, where $n=1$, 2, 3, ..., *N*, and *N* is the number of physical processes. $X_{in}$ measures how much process *n* controls the QoI. Similarly, $\textbf{Y}_j$ is *j*th RANS model’s feature vector, $Y_{jn}$ is the *n*th component in $\textbf{Y}_j$, and $Y_{jn}$ measures how well a RANS model handles the *n*th flow physical process.

The *j*th RANS model would work well for the *i*th QoI if their feature vectors align, and vice versa. Hence, $P_{ij}$ defined below measures if the *j*th RANS model would work well for the *i*th QoI

1 $$\begin{aligned} P_{ij}\equiv \sum _{n=1}^N{X}_{in}{Y}_{jn}. \end{aligned}$$

Equation (1) is called matrix factorization. We use $M_{ij}$ to denote an expert’s rating for the *i*th QoI and the *j*th RANS model. The error is measured as follows

2 $$\begin{aligned} E=\sum _{(i,j)\in \mathcal {O}}\left[ \sum _{n=1}^N{X}_{in}{Y}_{jn} - M_{ij} \right] ^2, \end{aligned}$$

where $\mathcal {O}$ contains (*i*, *j*) pairs for which $M_{ij}$ is available.

### Training

The objective of training is to find $X_{in}$ and $Y_{jn}$, $i=1, 2, 3..., I$, $j=1, 2, 3, ..., J$, $n=1, 2, 3, ..., N$ such that

3 $$\begin{aligned} (\textbf{X, Y}) = {\mathop {{\mathrm{arg\, min}}}\limits _{\textbf{X, Y}}} [E +\textrm{Constraints}], \end{aligned}$$

Equation (3) is called “collaborative filtering”. It is the process of filtering for information involving collaboration among multiple agents. In Eq. (3), collaborative filtering involves the QoIs, the models, and the data $M_{ij}$.

We elaborate on the “Constraints” in Eq. (3). $X_{in}$ measures how relevant the *n*th physics is in determining the *i*th QoI, and therefore $X_{in}$ is non-negative, and the sum of the $X_{in}$ from $n=1$ to *N* is 100%, i.e.,

4 $$\begin{aligned} \begin{aligned} \sum _{n=1}^N X_{in}&= 1, ~~~~i=1,..,I;\\ X_{ik}&\ge 0. \end{aligned} \end{aligned}$$

$Y_{jn}$ measures how the *j*th model captures the *n*th physics. Hence, $Y_{jn}$ is non-negative and less than 100%, i.e.,

5 $$\begin{aligned} 0\le Y_{jn}\le 1,~~~~j=1,..,J;\ n=1,...,N. \end{aligned}$$

A direct consequence of Eqs. (4) and (5) is that

6 $$\begin{aligned} 0<P_{ij} = \sum _{n=1}^N{X}_{in}{Y}_{jn} \le \sum _{n=1}^N X_{in} = 1. \end{aligned}$$

The rating is good, fair, or poor. Here, [0, 0.3333) corresponds to poor, [0.3333, 0.6667) corresponds to fair, and [0.6667, 1.0000] corresponds to good.

Due to the discretization process, the rating is not too sensitive to the change of $X_{in}$ and $Y_{jn}$. For example, if we already know the feature vector of the *j*th model $Y_{jn}$, we can evaluate a new QoI with feature vector $X_{in}$ with discrepancies, for the simplicity of analysis, $\Delta X_{in_1}$ and $\Delta X_{in_2}$ for the $n_1$th and $n_2$th physics only. According to Eq. (4), the discrepancies are of the same absolute value $\Delta X_{in_1}=-\Delta X_{in_2}=\Delta x_0$. Thus, the change of the prediction is $\Delta P_{ij}=\Delta x_0(Y_{jn_1}-Y_{jn_2})$. If we use the average of the range as the true value, the rating will not change when the error in prediction is less than 1/6. Even in the most extreme case, according to Eq. (5), the discrepancy can be as large as 1/6 while not affecting the correct rating, which is a fairly high tolerance level when considering the relative importance of the physical processes. Similar analysis can be done for $\textbf{Y}$, and we can see that $\textbf{Y}$ is even less sensitive.

The constraints in Eqs. (4), (5) are imposed by adding penalties in the cost function. That is

7 $$\begin{aligned} \begin{aligned} {\textrm{Constraint}}=&\lambda _1 \sum _{i=1}^I\left( \sum _{n=1}^N X_{in}-1\right) ^2+ \lambda _2\sum _{i=1}^I \sum _{n=1}^{N}\left( X_{in} - \left| X_{in}\right| \right) ^2 + \\&\lambda _3\sum _{j=1}^J \sum _{n=1}^{N}\left( Y_{jn} - \left| Y_{jn}\right| \right) ^2 + \lambda _4\sum _{j=1}^{J}\sum _{n=1}^N \left( 1 - Y_{jn} - \left| 1 - Y_{jn}\right| \right) ^2, \end{aligned} \end{aligned}$$

where $\lambda _{k}>0$, $k=1$, 2, 3, 4 is the penalty parameter. These are rather hard constraints, and we set $\lambda _k\equiv 1000$, i.e., a large number. The first two terms penalize the cost function if Eq. (4) is violated, while the last two terms penalize the cost function if (5) is violated. The matrix $\textbf{X}$ is regularized as follows: we set any $X_{in}$ to 0 if it is much smaller than the other components in the vector $\textbf{X}_i$. We then linearly re-scale the other components such that they satisfy Eq. (4).

### Initialization

We assign initial values of $\textbf{X}$ and $\textbf{Y}$ using our knowledge of the RANS model and the QoI. We note that if we have a lot of data, we can initialize $\textbf{X}$ and $\textbf{Y}$ randomly and still arrive at a physical solution. However, since data is always limited, a good initial guess is critical to the performance of a machine learning model^26^. For example, consider the prolate spheroid case, where the velocity at the location of separation is the quantity of interest. It should be clear that separation plays a significant role in determining the velocity there. Wall and free shear play a secondary role. It follows that [0, 0.1, 0.9] is a good initial condition, so as [0.1, 0.1, 0.8], [0.05, 0.1, 0.85], etc.

### RANS codes and data collection

A RANS calculation solves the Reynolds averaged Navier–Stokes equation. Due to the ensemble averaging, there are unclosed terms in the RANS equations, and they must be closed through modeling. We limit ourselves to sublayer-resolved closures for simplicity and consider the following RANS models: the one-equation Spalart-Allmaras (SA) model^27^, two two-equation $k-\epsilon$ models^28,29^, the standard two-equation $k-\omega$ model^30^ and its shear-stress transport (SST) variant^31^, the two-equation $k-kL$ model^32^, an explicit algebraic stress $k-\omega$ model (EASM)^33^, and two seven-equation full Reynolds stress models (RSM)^34,35^.

Three codes are used for the RANS calculations. NPHASE-PSU^14^ is an in-house research code. STAR-CCM+^15^ is a commercial code. We also make use of the TMR repository. The data there was generated using the NASA code CFL3D. CFL3D^16^ is an open-source NASA code. NPHASE-PSU and STAR-CCM+ are unstructured solvers, and CFL3D uses structured meshes. The same block-structured meshes are used for all three codes. These meshes are readily available on the TMR website. We run all three codes at appropriate Mach numbers for the test cases at hand. For each flow, we impose the same boundary conditions as provided by the TMR website.

Further details of these cases, including geometry, boundary condition specifics, and citations to comparison data sets, can be found there and in Ref.^36^. We also study a 3D prolate spheroid test case^37^.

In all, nine RANS turbulence models and ten QoIs are considered. 72 RANS analyses are conducted using two codes: an in-house code, NPHASE-PSU, and the commercial code, STAR-CCM+; another 40 RANS analyses are obtained from the TMR website for CFL3D.

## Supplementary Information

Supplementary Information.

## Data Availability

Raw data are available from the corresponding author.
