# Supplementary material for: Distilling experience into a physically interpretable recommender system for computational model selection
Source: Sci Rep. 2023 Feb 8;13:2225. doi: 10.1038/s41598-023-27426-5 (PMC9908871; doi:10.1038/s41598-023-27426-5)
Supplement: Supplementary file 1 — Supplementary Information. [file 41598_2023_27426_MOESM1_ESM.pdf]

Distilling experience into a physically interpretable recommender system for computational model selection

Xinyi Huang, Thomas Chyczewski, Zhenhua Xia, Robert Kunz, Xiang Yang

<table>
<tr><th></th><th colspan="2">C_f
2DZP</th><th colspan="2">C_p
ASBL</th><th colspan="2">U
2DML</th><th colspan="2">C_L
2DN00</th><th colspan="2">U
ASJ</th><th colspan="2">C_p
2DBFS</th><th colspan="2">C_p
ATB</th><th colspan="2">C_p
2DCC</th><th colspan="2">U_{L1}
PS</th><th colspan="2">U_{L2}
PS</th></tr>
<tr><td rowspan="2">(N) SA</td><td colspan="2">3</td><td colspan="2">2</td><td colspan="2">3</td><td colspan="2">3</td><td colspan="2">2</td><td colspan="2">1</td><td colspan="2">2</td><td colspan="2">3</td><td colspan="2">1</td><td colspan="2">1</td></tr>
<tr><td>3</td><td>3</td><td>2</td><td>2</td><td>2</td><td>3</td><td>3</td><td>3</td><td>2</td><td>2</td><td>2</td><td>2</td><td>2</td><td>2</td><td>3</td><td>3</td><td>1</td><td>1</td><td>1</td><td>1</td></tr>
<tr><td rowspan="2">(N) $k-\epsilon$
Chien</td><td colspan="2">3</td><td colspan="2">1</td><td colspan="2">3</td><td colspan="2">1</td><td colspan="2">2</td><td colspan="2">3</td><td colspan="2">1</td><td colspan="2">3</td><td colspan="2">1</td><td colspan="2">1</td></tr>
<tr><td>3</td><td>3</td><td>1</td><td>1</td><td>3</td><td>3</td><td>2</td><td>1</td><td>2</td><td>2</td><td>2</td><td>2</td><td>1</td><td>1</td><td>3</td><td>3</td><td>1</td><td>1</td><td>1</td><td>1</td></tr>
<tr><td rowspan="2">(N) $k-\omega$
SST</td><td colspan="2">3</td><td colspan="2">3</td><td colspan="2">1</td><td colspan="2">3</td><td colspan="2">2</td><td colspan="2">2</td><td colspan="2">3</td><td colspan="2">3</td><td colspan="2">3</td><td colspan="2">3</td></tr>
<tr><td>3</td><td>3</td><td>3</td><td>3</td><td>1</td><td>1</td><td>3</td><td>3</td><td>2</td><td>2</td><td>2</td><td>2</td><td>3</td><td>3</td><td>3</td><td>3</td><td>3</td><td>3</td><td>3</td><td>3</td></tr>
<tr><td rowspan="2">(N) $R_{ij}-\omega$</td><td colspan="2">3</td><td colspan="2">-</td><td colspan="2">-</td><td colspan="2">-</td><td colspan="2">-</td><td colspan="2">-</td><td colspan="2">2</td><td colspan="2">-</td><td colspan="2">-</td><td colspan="2">-</td></tr>
<tr><td>-</td><td>-</td><td>-</td><td>-</td><td>-</td><td>-</td><td>-</td><td>-</td><td>-</td><td>-</td><td>-</td><td>-</td><td>-</td><td>-</td><td>-</td><td>-</td><td>-</td><td>-</td><td>-</td><td>-</td></tr>
<tr><td rowspan="2">(T) SA</td><td colspan="2">3</td><td colspan="2">2</td><td colspan="2">3</td><td colspan="2">3</td><td colspan="2">2</td><td colspan="2">1</td><td colspan="2">1</td><td colspan="2">3</td><td colspan="2">-</td><td colspan="2">-</td></tr>
<tr><td colspan="2">3</td><td colspan="2">2</td><td colspan="2">3</td><td colspan="2">3</td><td colspan="2">2</td><td colspan="2">1</td><td colspan="2">1</td><td colspan="2">3</td><td colspan="2">-</td><td colspan="2">-</td></tr>
<tr><td rowspan="2">(T) $k-\omega$
2006</td><td colspan="2">3</td><td colspan="2">3</td><td colspan="2">1</td><td colspan="2">3</td><td colspan="2">-</td><td colspan="2">2</td><td colspan="2">2</td><td colspan="2">3</td><td colspan="2">-</td><td colspan="2">-</td></tr>
<tr><td colspan="2">3</td><td colspan="2">3</td><td colspan="2">1</td><td colspan="2">3</td><td colspan="2">3</td><td colspan="2">2</td><td colspan="2">2</td><td colspan="2">3</td><td colspan="2">-</td><td colspan="2">-</td></tr>
<tr><td rowspan="2">(T) $k-\omega$
SST</td><td colspan="2">3</td><td colspan="2">3</td><td colspan="2">2</td><td colspan="2">3</td><td colspan="2">3</td><td colspan="2">2</td><td colspan="2">2</td><td colspan="2">3</td><td colspan="2">-</td><td colspan="2">-</td></tr>
<tr><td colspan="2">3</td><td colspan="2">3</td><td colspan="2">2</td><td colspan="2">3</td><td colspan="2">3</td><td colspan="2">2</td><td colspan="2">2</td><td colspan="2">3</td><td colspan="2">-</td><td colspan="2">-</td></tr>
<tr><td rowspan="2">(T) $k-kL$</td><td colspan="2">3</td><td colspan="2">-</td><td colspan="2">2</td><td colspan="2">3</td><td colspan="2">-</td><td colspan="2">3</td><td colspan="2">1</td><td colspan="2">-</td><td colspan="2">-</td><td colspan="2">-</td></tr>
<tr><td colspan="2">3</td><td colspan="2">3</td><td colspan="2">2</td><td colspan="2">3</td><td colspan="2">3</td><td colspan="2">2</td><td colspan="2">2</td><td colspan="2">3</td><td colspan="2">-</td><td colspan="2">-</td></tr>
<tr><td rowspan="2">(T) EASM</td><td colspan="2">3</td><td colspan="2">2</td><td colspan="2">1</td><td colspan="2">3</td><td colspan="2">-</td><td colspan="2">1</td><td colspan="2">-</td><td colspan="2">3</td><td colspan="2">-</td><td colspan="2">-</td></tr>
<tr><td colspan="2">3</td><td colspan="2">2</td><td colspan="2">1</td><td colspan="2">3</td><td colspan="2">2</td><td colspan="2">1</td><td colspan="2">1</td><td colspan="2">3</td><td colspan="2">-</td><td colspan="2">-</td></tr>
<tr><td rowspan="2">(T) $R_{ij}-\omega$</td><td colspan="2">3</td><td colspan="2">3</td><td colspan="2">2</td><td colspan="2">3</td><td colspan="2">-</td><td colspan="2">-</td><td colspan="2">2</td><td colspan="2">3</td><td colspan="2">-</td><td colspan="2">-</td></tr>
<tr><td colspan="2">3</td><td colspan="2">3</td><td colspan="2">2</td><td colspan="2">3</td><td colspan="2">3</td><td colspan="2">2</td><td colspan="2">2</td><td colspan="2">3</td><td colspan="2">-</td><td colspan="2">-</td></tr>
</table>

Supplementary Table S1: Ratings of all RANS models for all QoIs. We use “N” and “T” to denote the NPHASE-PSU results, and the results on the TMR results. Here, 3 is good, 2 is fair, and 1 is poor. The first row of each model is the training data, and the second row are the predictions. The predictions for NPHASE-PSU results are split into 2 columns. The first column is the ratings from BRS, while the second column is the ratings from ARS. The prediction ratings for TMR website results are from BRS only. The predictions are red if they are different from the data.

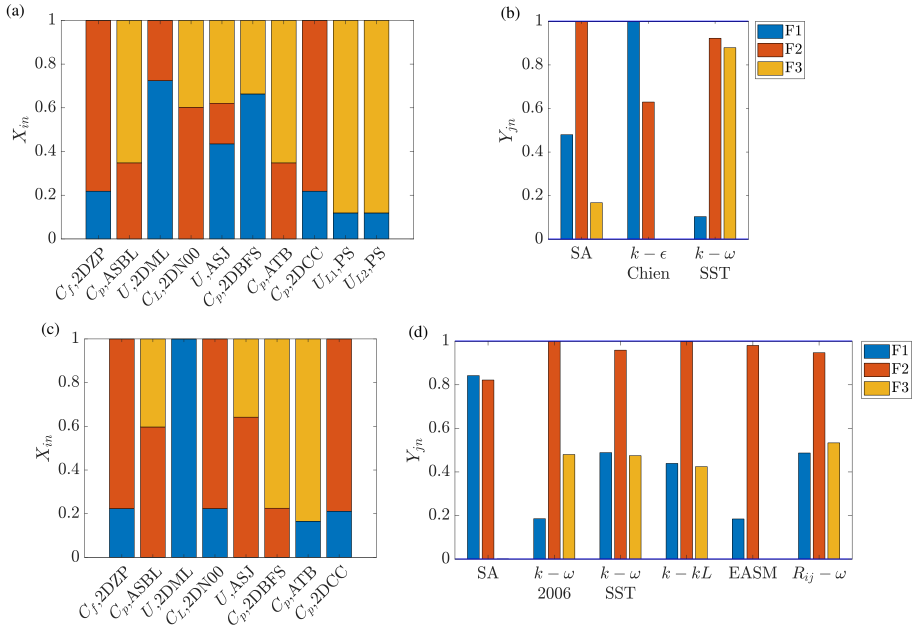

Supplementary Figure S1: A visualization of (a,c) QoIs' feature vectors, $\mathbf{X_i}$ and (b,d) models' feature vectors, $\mathbf{Y_j}$ for (a,b) BRS-N and (c,d) BRS-T.

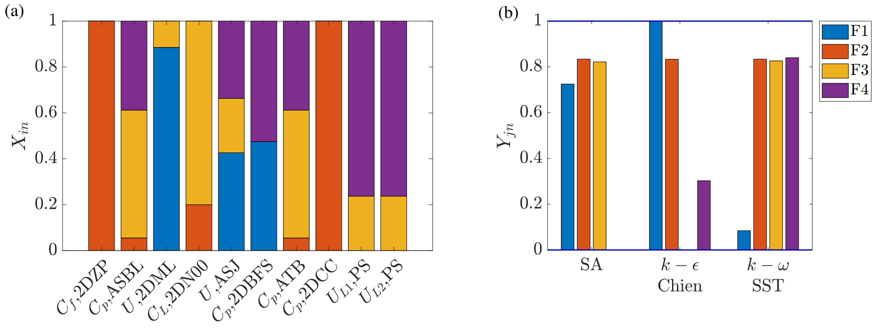

Supplementary Figure S2: Same as figure S1 but for ARS-N, where the feature number $N = 4$.

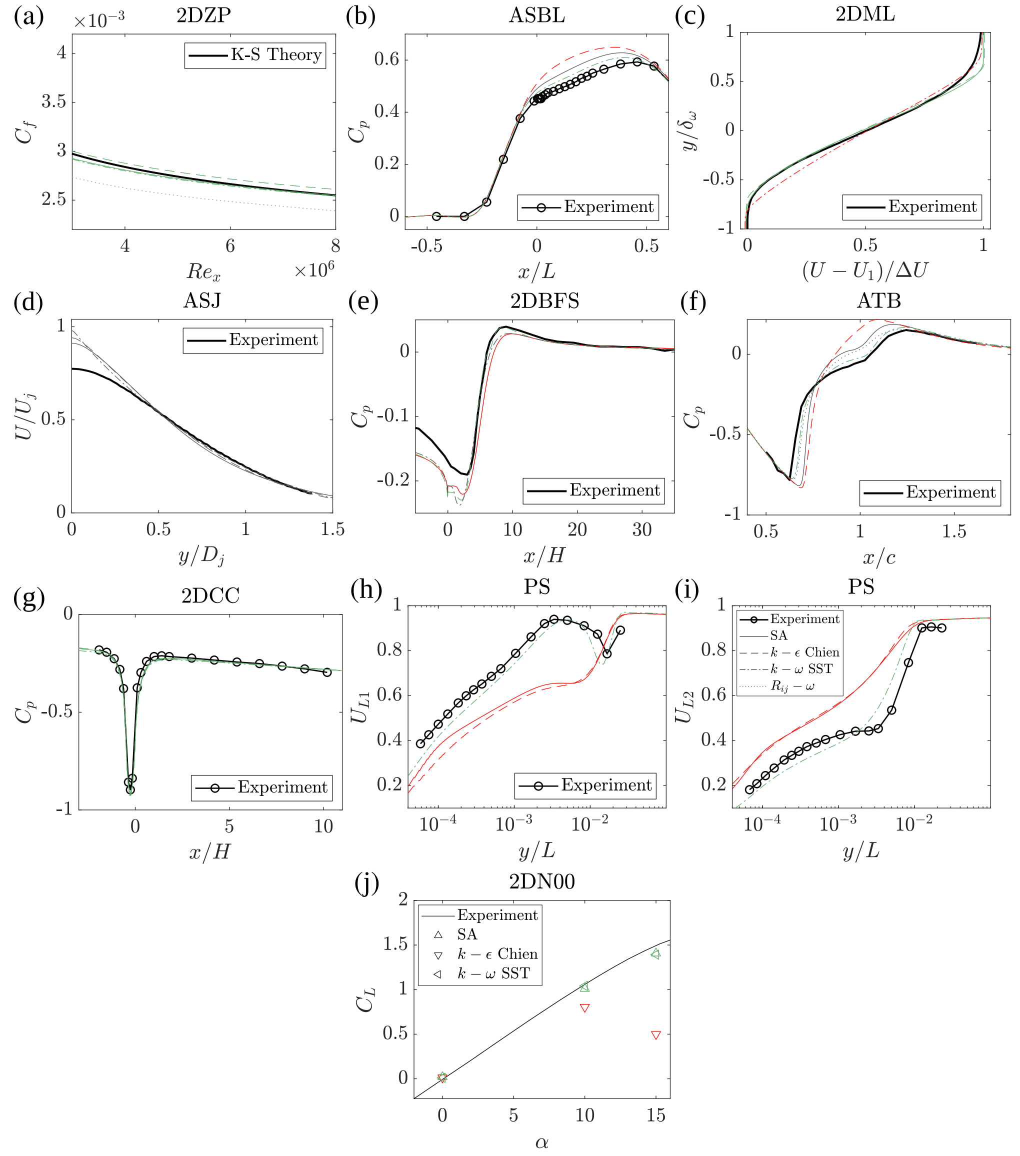

Supplementary Figure S3: NPHASE-PSU RANS results. (a-i) Thin lines are for the SA model, dashed lines are for the $k-\epsilon$ Chien model, dot-dashed lines are for the $k-\omega$ SST model, and dotted lines are for the $R_{ij}-\omega$ model. (j) The 2DN00 results, where different symbols are used for different RANS models. We use green for "good" results, gray for "fair" results, and red for "poor" results.

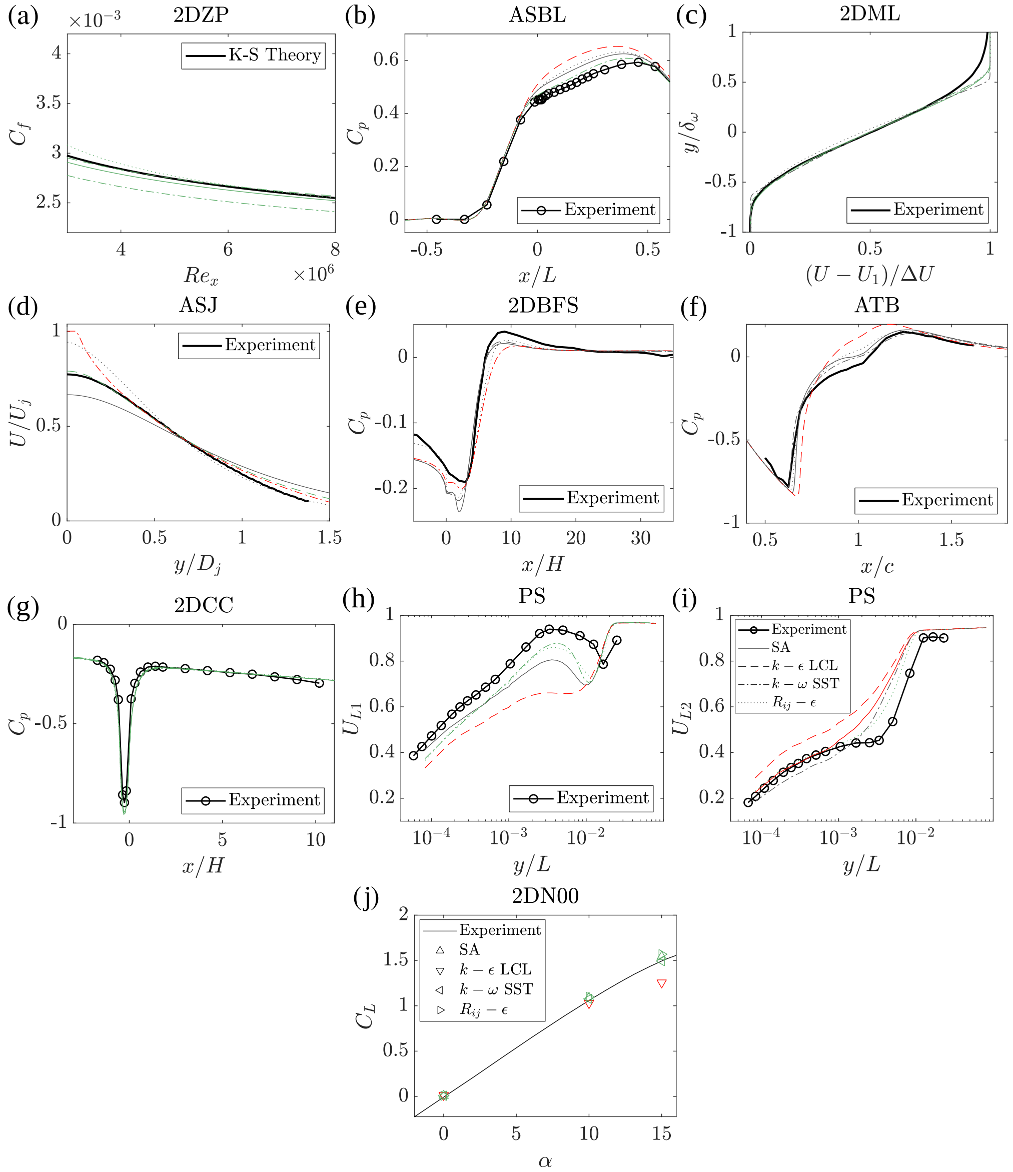

Supplementary Figure S4: STAR-CCM+ RANS results. (a-i) Thin lines are for SA model, dashed lines are for the $k-\epsilon$ LCL model, dot-dashed linesare for the $k-\omega$ SST model, and dotted lines are for the $R_{ij}-\epsilon$ model. (j) The 2DN00 results, where different symbols are used for different RANS models. We use green for "good" results, gray for "fair" results, and red for "poor" results.

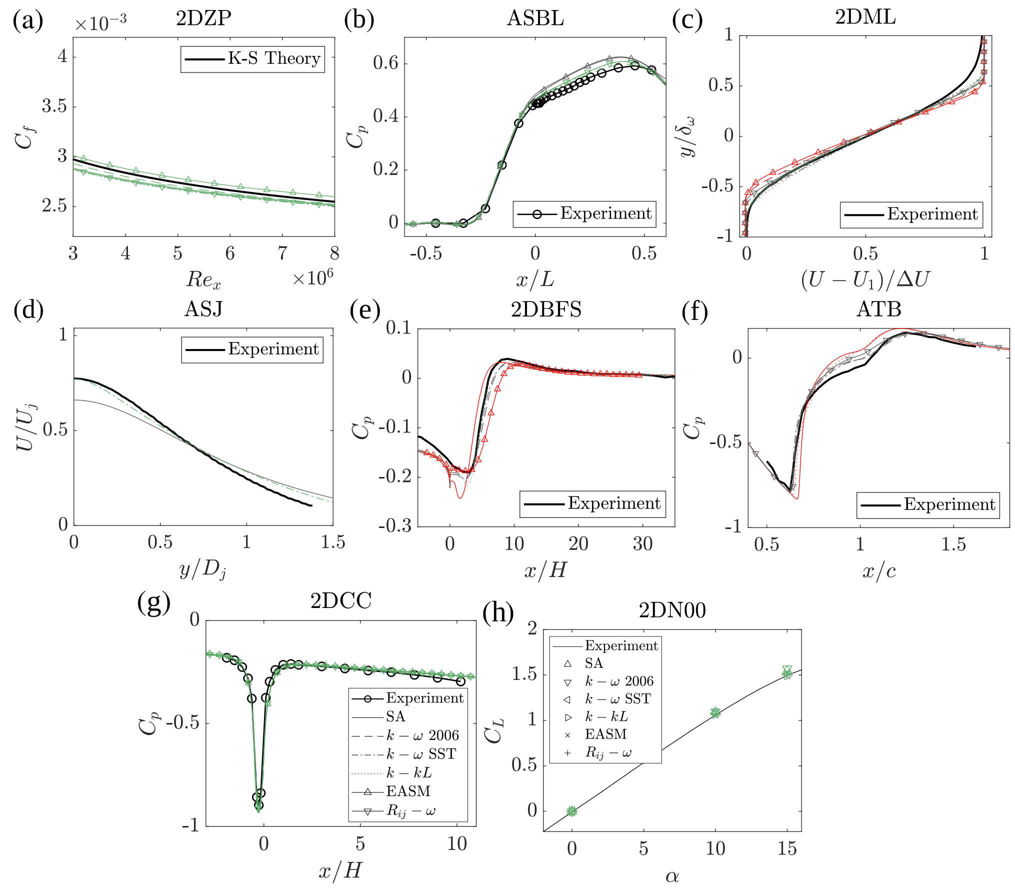

Supplementary Figure S5: TMR RANS results. (a-g) Thin lines are for the SA model, dashed lines are for the $k-\omega$ 2006 model, dot-dashed lines are for the $k-\omega$ SST model, dotted lines are for the $k-kL$ model, thin lines with upward point triangles are for the EASM model, and thin lines with downward point triangles are for the $R_{ij}-\omega$ model. (h) The 2DN00 results, where different symbols are used for different RANS models. We use green for "good" results, gray for "fair" results, and red for "poor" results.
